# Breast cancer metastatic to the pituitary gland: a case report

**DOI:** 10.1186/1477-7819-10-137

**Published:** 2012-07-09

**Authors:** Gian Paolo Spinelli, Giuseppe Lo Russo, Evelina Miele, Natalie Prinzi, Federica Tomao, Manila Antonelli, Felice Giangaspero, Valeria Stati, Martina Strudel, Silverio Tomao

**Affiliations:** 1 Department of Medico-Surgical Sciences and Biotechnologies, University of Rome "Sapienza," - Distretto ASL di Aprilia - Via Giustiniano snc, 04011, Aprilia, LT, Italy; 2 Department of Molecular Medicine, University of Rome ‘Sapienza’, Viale Regina Elena 324, 00161, Rome, Italy; 3 Department of Experimental Medicine, University of Rome ‘Sapienza’, Viale Regina Elena 324, 00161, Rome, Italy; 4 Department of Gynaecology and Obstetrics, University of Rome "Sapienza", Viale Regina Elena 324, 00161, Rome, Italy; 5 Department of Radiological, Pathological and Oncological Sciences Neurological Sciences, University of Rome ‘Sapienza’, Viale Regina Elena 324, 00161, Rome, Italy

**Keywords:** Breast cancer, Pituitary metastases, Follow-up, Controversial diagnosis

## Abstract

**Background:**

Metastases to the pituitary gland are rare events, and usually indicate widespread malignant disease. The lung and the breast are the most common sites of primary tumors that metastasize to the pituitary gland.

Metastases are more frequent in older patients and the most common symptoms at presentation are diabetes insipidus and visual alterations.

**Case presentation:**

72-year-old white woman was treated for a breast carcinoma with right superoexternal quadrantectomy, radiotherapy, and hormone therapy. Twelve years later, the patient presented with bone pain, bilateral progressive visual decline, and onset of hypopituitarism. A diagnosis of secondary bone involvement and pituitary metastasis was made.

**Conclusion:**

This was an unusual disease course, and stresses the importance of intensive follow-up in patients with breast cancer even many years after the initial diagnosis This case emphasizes that diagnosis can be difficultand controversial when relapse occurs at uncommon sites.

## Background

Pituitary metastases are an uncommon complication of cancer, representing only 1% of the pituitary lesions [1,2] and 0.14% to 28% of all brain metastases in reported autopsy series [3,4]. They are often related to primary breast (20% to 30% of cases) or lung cancers (30% to 50% of cases), with other sites of primary tumors being reported less often [1,2,5,6]. Pituitary metastases are symptomatic in only 8% of cases [1,2], with the most important signs being diabetes insipidus and anterior pituitary gland dysfunction. The tumor mass may also cause headache, visual alterations, and ophthalmoplegia [1,2,5,6]. No gender predominance has been reported [2]. At diagnosis, most patients are elderly, and have widespread disease with multiple sites of implantation [7,8]. Pituitary metastases may also be the first presentation of an occult primary cancer or may be the only site of metastasis [9-11]. They very rarely occur in early adulthood [12]. The rarity and the lack of specific radiological and clinical signs make it difficult to distinguish these tumors from other more common benign pituitary lesions [9,13]. We present a case of late recurrence of breast cancer presenting asbone metastases and a pituitary metastatic mass.

## Case presentation

A 72 year-old woman underwent a superoexternal quadrantectomy with axillary lymph-node dissection for an infiltrating ductal carcinoma of the right breast (not otherwise specified variant, G1, pT1c, pN0, estrogen receptor-positive, progesterone receptor-positive), and was treated with radiotherapy and tamoxifen for 5 years. There were no signs of recurrence for almost 12 years, at which point gait disturbances appeared.

Total body computed tomography showed numerous hyperdense lesions in many vertebral bodies and costal arches. A bone scan was performed, but the lesions were not confirmed as metastases. Despite the negative results for bone scan and tumor markers, a bone biopsy was taken, and the results of this examination led to a diagnosis of metastatic disease. The patient was started on treatment with letrozole.

Around 4 months later, the patient began experiencing visual disturbances. Magnetic resonance imaging (MRI) of the pituitary region showed a sellar mass, approximately 11 mm in diameter, compressing the optic chiasm. The Lesion was compatible with pituitary adenoma, which was confirmed by endocrinologica findings documenting the presence of hypopituitarism. Laboratory test results showed that the patients thyroid function, gonadotropin and serum prolactin levels were normal, but there was hyponatremia and hyperkalemia and reduced levels of adrenocorticotropic hormone and cortisol. The patient was started on steroid therapy.

**Figure 1:**
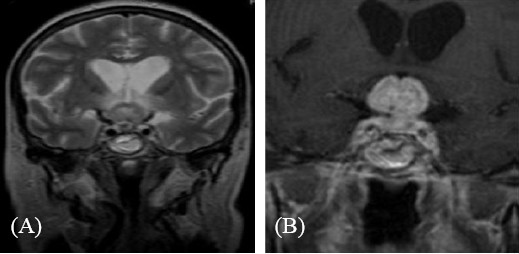
**T1-weighted magnetic resonance imaging scan of the brain.** (**A**) Coronal image of pituitary metastasis causing compression of the optic chiasm; (**B**) detail of optic chiasm compression.

**Figure 2:**
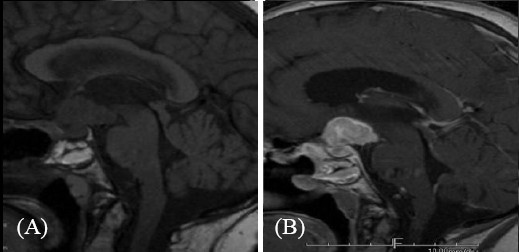
**T1-weighted magnetic resonance imaging.** (**A**) Pre-contrast mid-sagittal image of pituitary metastasis; (**B**) post-contrast detail of pituitary metastasis.

**Figure 3:**
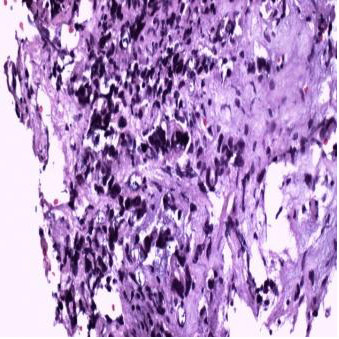
**Microscopic examination.** Neoplasm composed of atypical cells with hyperchromatic and anaplastic nuclei.

Three months later, owing to progressive deterioration of the visual disturbances (bi-temporal hemianopsia), gadolinium-enhanced MRI of the brain was carried out, which showed a large heterogeneous mass, which appeared intensely opaque after contrast. Moreover, the mass had a supra-sellar extension and was infiltrating the optic chiasm (Figure 1, Figure 2). Despite the probable metastatic nature of the lesion, the patient underwent a trans-sphenoidal surgery of the tumor and a subtotal resection was achieved. The histological findings identified the mass as a malignant neoplasm compatible with metastatic breast cancer and specifically a poorly differentiated adenocarcinoma (Figure 3). The immunohistochemistry results showed that the cells were positive for estrogen and progestin receptor and negative for ErbB-2, Unfortunately, the surgery did not improve the patient’s visual disturbances, and no other treatments were performed because of the rapid deterioration in her clinical condition.

## Discussion

Breast cancer is one of the most common types of cancer, and recurrence within 5 years after diagnosis is frequent; however, rates of recurrence and disease relapse have greatly improved over the past 10 years is not common. In general, the most important sites for metastasis are bone, liver, and lung [14].

The pituitary gland is a rare site for metastasis for all neoplasm (metastases make up less than 1% of pituitary tumors) [1,2]. Therefore, the clinical history of our patient shows a rare pattern of breast cancer metastasis. However, cancers of almost all tissues can metastasize to the pituitary gland.

The lung and breast are the most important locations for the primary tumor localizations in men and women, respectively, but in about 5% of cases the primary cancer remains unknown [1,2,5-7]. Identification of metastases is more common in elderly patients, and in many cases is suggestive of the presence of disseminated disease and carries a poor prognosis[7,8]. The hematogenous spread (direct to the pituitary parenchyma or diaphragm sellae through the portal vessels) is the most important mechanism for development of these metastases. Alternative hypotheses for spread are extension from an adjacent bone metastasis or a meningeal spread through the supra-sellar cistern [7,10]. The posterior lobe is more affected than the anterior lobe, mainly because of the lack of a direct arterial blood supply to the anterior lobe and because there is a larger area of contact between the posterior lobe and the adjacent *dura madre*[3,15]. For this reason, the most common sign of this metastatic involvement is diabetes insipidus [1,2,4,13], whereas hypoadrenalism and bilateral hemianopsia, as seen in our patient, are less common [1,2].

The case shows that the metastatic lesions of pituitary gland can mimic the signs and symptoms of pituitary macroadenoma, leading to a delay in diagnosis of several months. The rarity of this event made diagnosis and the subsequent choice of therapeutic approach difficult.

## Conclusion

We report this rare clinical case is primarily to emphasize the importance of careful follow-up, especially in breast cancer. The correct differential diagnosis between benign and malignant lesions in the pituitary region is essential for effective treatment.

## Consent

Written consent was obtained from the patient for publication of this case report.

## Competing interests

The authors declare that they have no competing interests.

## Authors’ contributions

GPS, GLR, EM and ST conceived of the study. NP, FT, VS, and MS participated in study design and clinical data collection. MA and FG carried out the histopathological evaluation. GPS, GLR and EM drafted the manuscript. All authors read and approved the final manuscript.
